# Implementation of remote asthma consulting in general practice in response to the COVID-19 pandemic: an evaluation using extended Normalisation Process Theory

**DOI:** 10.3399/BJGPO.2021.0189

**Published:** 2022-01-26

**Authors:** Jonathan Stewart, Noleen McCorry, Helen Reid, Nigel Hart, Frank Kee

**Affiliations:** 1 Centre for Public Health, Queen’s University Belfast, Belfast, UK; 2 Centre for Medical Education, Queen's University Belfast, Belfast, UK

**Keywords:** asthma, qualitative research, remote consulting, COVID-19, general practice, primary healthcare, telemedicine, telehealth

## Abstract

**Background:**

The COVID-19 pandemic has led to the rapid and reactive deployment of remote consulting in UK general practice. The delivery of acute and chronic asthma care has been affected. Extended Normalisation Process Theory (eNPT) provides a framework for evaluating the implementation of new complex interventions in routine practice, including examination of how context–intervention interactions affect implementation.

**Aim:**

To explore the implementation of remote asthma consulting in UK general practice in response to the COVID-19 pandemic.

**Design & setting:**

Mixed-methods evaluation, which was informed by eNPT, in general practice in Northern Ireland.

**Method:**

Data were collected from a range of healthcare professionals who provide asthma care using online questionnaires, interviews, and multidisciplinary focus groups. Analysis was informed by eNPT.

**Results:**

Ten themes were identified to describe and explain the contribution of general practice staff to implementation of remote asthma consulting. Staff identified novel alternatives to in-person review. Having a practice champion to drive implementation forward, and engage other practice staff, was important. Patient, staff, and healthcare system-contextual factors influencing implementation were identified including access to, understanding of, and willingness to use the technology required for remote consulting.

**Conclusion:**

The experiences of frontline healthcare professionals in this study indicate that remote asthma consulting has potential benefits in terms of access and effectiveness when implementation integrates seamlessly with face-to-face care for those who want or need it. Work is required at practice and healthcare system levels to realise this potential, and ensure implementation does not exacerbate existing healthcare inequalities.

## How this fit in

The COVID-19 pandemic has led to the rapid and reactive deployment of remote consulting in UK general practice. For asthma, the current evidence base for remote consulting is limited. The experiences of frontline healthcare professionals in this study demonstrate ‘work’ that can be done at staff, practice, and healthcare system-levels to support implementation and integration of remote asthma consulting into everyday clinical practice.

## Introduction

Provision of asthma care has been dramatically affected by the COVID-19 pandemic. In March 2020, to reduce viral spread, general practices in the UK were advised to consult remotely with patients where possible. Patients deemed at highest risk from COVID-19 were advised to ‘shield’ themselves, including patients with severe asthma.^1^ For acute asthma care, interim guidance advised that, where possible, following risk assessment and determination of severity, mild and low-risk moderate asthma exacerbations could be managed remotely during the pandemic period.^2–5^ Chronic asthma care has also been affected. The majority of chronic asthma care in the UK is delivered in general practice. The Quality and Outcomes Framework (QOF), the key method of remuneration for chronic asthma care in general practice, was paused at the start of the pandemic to shift resources and focus to acute care.

The current evidence base for remote asthma consulting (RAC) is limited. When compared with face-to-face consultations, a previous randomised controlled trial found telephone consultations enabled more people with asthma to be reviewed, without clinical disadvantage, or loss of satisfaction.^6^ A previous systematic review of RAC investigated its use in addition to, rather than instead of, face-to-face consulting, and concluded that current evidence did not support widespread implementation of additional remote visits.^7^

Asthma consultations can be considered complex interventions, meaning (among other things) that they are composed of multiple interacting components.^8^ Consulting remotely adds a further layer of complexity. Normalisation Process Theory (NPT) is a mid-range theory, which identifies factors that promote and inhibit the routine incorporation of complex interventions into everyday practice.^9^ NPT predicts that for any new way of working to become fully embedded into everyday practice, continuous work is required in four domains of activity: sense-making (coherence), engagement, action, and monitoring (Table 1). NPT has developed over time, with the most recent iteration of the framework, extended NPT (eNPT), enabling examination of how context–intervention interactions affect implementation.^10^

**Table 1.** Extended NPT domains

**Contribution**	Implementation of RAC depends on people's continuous contributions to enacting it by investing in: Coherence: making sense of the reasons for RAC (purpose and possibilities) Engagement: buy-in to and engagement with RAC Action: putting RAC into action Monitoring: appraising the impact of the move to RAC
**Capability**	The capability of people to enact RAC depends on its workability and integration into everyday practice.
**Capacity**	The incorporation of RAC into its clinical context depends of the capacity of people to cooperate and coordinate their actions.
**Potential**	The translation of potential into action depends on people’s intentions to enact the intervention and their potential to build shared commitments with other professionals.

NPT = Normalisation Process Theory. RAC = remote asthma consulting.

This study aimed to evaluate the rapid and reactive implementation of RAC in general practice in response to the COVID-19 pandemic, through the lens of eNPT, to identify factors that promoted or inhibited implementation, and identify the ‘work’ that is required for ‘normalisation’ into routine clinical care.

## Method

### Study design

A mixed-methods evaluation of the implementation of remote asthma consulting (RAC) was conducted, comprising an online questionnaire, semi-structured interviews, and focus groups. The study was informed by eNPT.

A broad definition of RAC was adopted, including telephone, video, or patient messaging. The study setting was general practice in Northern Ireland. In June 2021 Northern Ireland had 321 active GP practices, and a total of 2 007 000 individuals registered with a GP practice.^11^ Study participants were members of general practice multidisciplinary teams in Northern Ireland who provide asthma care including GPs, GP trainees, nurses, and pharmacists. Team members with experience of acute or chronic asthma consulting were included, with the aim of learning how the spectrum of asthma care had been implemented remotely.

### Data collection

#### Online questionnaire

An online questionnaire based on the Normalisation MeAsure Development questionnaire (NoMAD) tool^12^ was disseminated to general practice staff who provide asthma care across Northern Ireland. NoMAD is a NPT-informed 23-item instrument for measuring implementation processes from the perspective of professionals directly involved in the work of implementing complex interventions, and was adapted for RAC.^12^ Responses are based on the 7-point Likert scale (strongly agree, agree, neither agree nor disagree, disagree, strongly disagree, not relevant to my role, and not relevant at this stage). The questionnaire was disseminated to staff via regional GP, GP trainee, practice-based pharmacist, and practice manager networks to facilitate representation from a sample of participants from across the region.

#### Semi-structured interviews

The final question of the online questionnaire invited responders to take part in a semi-structured interview to explore their experience of RAC further. Interviews were conducted with a purposive sample of practice staff based on job role until data saturation. The interview schedule was designed to cover the NPT domains.

#### Focus groups

Following analysis of the questionnaire and interview data, two virtual focus groups were conducted. A purposive sample of staff with known experience of RAC via clinical and academic networks was recruited to ensure representation of a range of multidisciplinary team members from across the region. The purpose of the focus groups was to further explore interview findings with general practice staff who deliver asthma care and to sense-check the most plausible programme theory for the deployment of RAC.

Interviews and focus groups were facilitated, digitally recorded, and transcribed by one researcher. Transcription data were transferred to NVivo (version 12) for analysis.

### Data analysis

Responses to the NOMAD online questionnaire were summarised using percentages across the 7-point Likert scale (Supplementary Figure 1). Interpretation of qualitative data was informed by framework analysis.^13^ Interview data were initially coded using the four core NPT domains (coherence, engagement, action, and monitoring). The framework was then expanded to include the extended NPT domains (capability, capacity, and potential) to analyse how context–intervention interactions affect implementation (Table 1). Interview data were coded by one researcher (JS), with three transcripts also independently coded by a second researcher (NM). Coding was compared and any disagreements discussed. The final framework was reviewed by a third researcher (HR) before being applied to the full interview dataset and NOMAD questionnaire data. The same framework was subsequently applied to the focus group data. Throughout analysis there was ongoing collaboration and discussion between researchers to ensure coding reliability and transparency. The study was reported according to the consolidated criteria for reporting qualitative research (COREQ) standards.^14^

## Results

**Figure 1.**
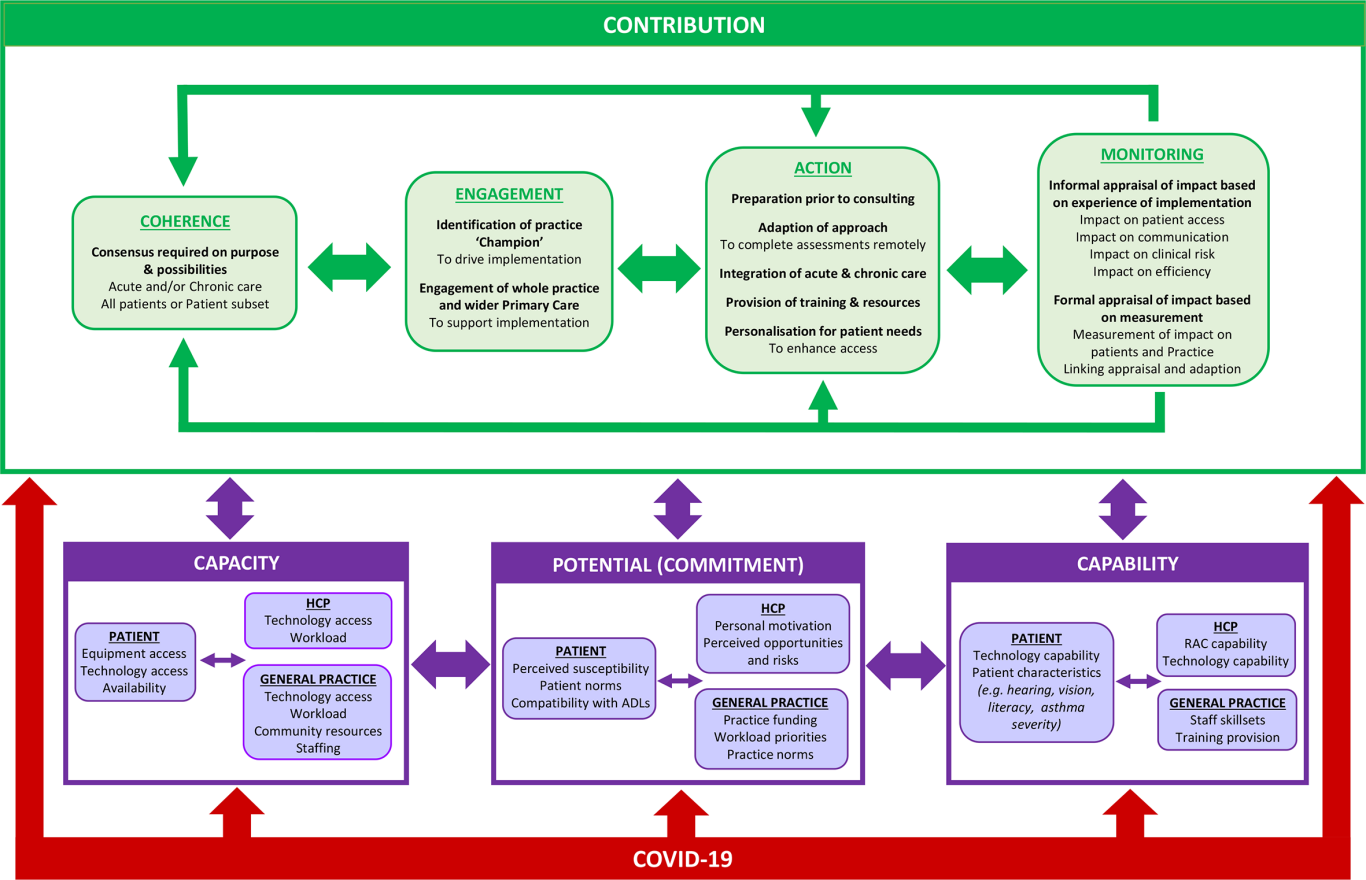
Summary of findings: work by members of the general practice team during implementation of remote asthma consulting in response to the COVID-19 pandemic, and contextual factoring impacting implementation. ADLs = activities of daily living. HCP = healthcare professional. RAC = remote asthma consulting.

The study describes the work by members of the general practice team during implementation of RAC and factors that impacted on implementation (Figure 1 and Supplementary Tables 1a–d). The qualitative and quantitative findings are presented in an integrated manner under the four domains of eNPT.

The questionnaire was completed by 63 general practice staff (12 GPs, 13 GP trainees, 11 nurses, 21 pharmacists, and six unknown) (Supplementary Figure 1). Experiences of implementation were explored further with nine questionnaire responders through qualitative interviews (three GPs, two GP trainees, one nurse, and three pharmacists). Following analysis of questionnaire and interview data, two focus groups were conducted (focus group 1: one GP, three nurses, and two pharmacists; focus group 2: one GP, one nurse, and one pharmacist).

### Contribution

Contribution describes how implementation of RAC depends on people's continuous investment in coherence, engagement, action, and monitoring. Ten themes were identified across these four core NPT domains to describe and explain the continuous ‘contribution’ of staff to implementation of RAC.

#### Contribution: coherence. Consensus required on purpose and possibilities

For successful implementation of a complex intervention, staff need to make sense of its purpose and possibilities. While the majority of questionnaire responders felt staff in their practice had a shared understanding of why RAC was required (92% agreed or strongly agreed), qualitative data indicated that staff coherence on the purpose and possibilities of RAC varied. There was general consensus among staff that RAC supported the ongoing delivery of acute asthma care within pandemic restrictions. However, physical examination presented a challenge for implementation. Beyond pandemic restrictions, some staff felt the need for examination could be identified during initial remote consulting, while others felt the inability to perform physical examination remotely limited the usefulness of RAC for acute asthma care:

*'* *I think we should continue with telephone triage before face-to-face review ... I don’t think half of the people who come in for face-to-face review need to come in.* *'* (INT4)

*'* *In terms of acute asthmatics, it is very difficult to beat seeing them face to face.* *'* (INT6)

For chronic asthma care, while some staff thought the components of a chronic asthma review were amendable to remote consulting, others questioned the accuracy of some of the required assessments, including inhaler technique. There were also concerns about the impact of RAC on access to spirometry, the current gold standard for new asthma diagnoses.

*'* *For chronic I think remote consulting is a good idea. I think you can cover most things over the phone.* *'* (INT4)

*'* *Inhaler use is important to me and that is something I do miss through the virtual arena. You can miss so much.* *'* (INT2)

There was also lack of consensus on whether RAC should be utilised for all patients or a subset. Staff agreed that some patients may benefit less from RAC, including those with poor access to required technology or more complex asthma. However, there was lack of consensus on if and how patients should be selected for remote consulting.

#### Contribution: engagement. Identify a practice ‘champion’ to drive implementation

Staff identified the importance of having someone take a lead role on implementation and engaging others in the practice.

*'* *We find it is helpful to have a lead person for things. Because you get this concept of* *"bystander atrophy"* *where nobody really takes responsibility.* *'* (FG9)

This was often someone with existing experience of delivering chronic asthma care, who could apply this experience to adapt care processes for remote consulting. In some cases, the ‘champion’ was a GP partner who was in a strategic leadership position and could provide team members with required resources and training. Questionnaire responders were divided on whether they thought that there was a key person driving RAC forward in their practice (57% agreed or strongly agreed).

#### Contribution: engagement. Engagement of the whole practice and wider primary care team

Almost all questionnaire responders saw participating in RAC as a legitimate part of their role (97% agreed or strongly agreed). Interview participants highlighted the need to engage the wider practice and primary care team, including *'encouraging the late adopters in the practice'* (INT1). For successful implementation, roles should be identified for all team members, including important roles for administrative staff:

*'While some may have a more clinical role, everyone has a role to play.* *'* (INT4)

Some practices benefitted from support from the wider primary care team for provision of care and training, including from community pharmacy colleagues and GP federations.

#### Contribution: action. Preparation with the patient prior to consultation

Preparation with patients before remote consulting was felt to enhance efficiency, and enable the approach to be personalised to patient needs. In some practices administrative staff members contacted patients before the remote review to identify their preferred consulting mode and to ensure they had access to the required equipment (for example, peak flow meter):

'*They speak to the patients first and explain this is going to be a virtual review and would they like to do a video review.*' (INT5)

Some practices supported patients to collect data before the consultation including peak flow readings and questionnaire data on level of asthma control (for example, Asthma Control Test).

#### Contribution: action. Adaptation of approach to complete required assessments remotely

Staff employed a variety of strategies to adapt the required assessments of acute and chronic asthma reviews for remote consulting. Staff modified existing asthma consultation templates on practice IT systems for remote care. To compensate for the absence of visual cues and physical examination when telephone consulting, more detailed history-taking was required, and some practices utilised video consulting to provide visual cues:

*'* *With video we can visualise the patient. You learn a lot from seeing the patient.* *'* (FG5)

Video consulting was also used to support assessment of inhaler technique. Practices used various other approaches to support patients with inhaler technique, including online videos, instructions on inhaler prescriptions, and signposting to community pharmacy colleagues to demonstrate technique when a prescribed inhaler was dispensed.

Some participants reported that written materials (for example, asthma action plans and patient information sheets), which would previously have been handed to the patient, were now sent via patient messaging software or made available on the practice website.

#### Contribution: action. Provision of training and resources

Staff highlighted the benefits of training, including bespoke remote asthma consulting training:

*‘They have been very supportive. They encouraged me to do the training'* (INT5)

Training was provided by some practices to support administrative staff to play a greater role in remote asthma consulting.

#### Contribution: action. Personalised approach to patient needs

Some staff felt delivery could be personalised to patient needs and preferences based on information gained before and during the consultation (telephone, video, and/or face to face). One staff member provided the analogy of air travel to demonstrate how, if implemented effectively, with adequate support for patients and practices, RAC could benefit everyone, even those for whom it is not appropriate:

*'* *I always think of when you go to the airport. It was you booked online. Then it was put the sticker on the suitcase yourself. Of course, some people won’t be able to do it, but that’s OK because now there is no queue at the desk, so the person who can’t use the technology can go to the desk and get it sorted* *.'* (FG2)

#### Contribution: action. Integration of acute and chronic remote asthma consulting

UK guidance advises that patients should have a chronic asthma review within 2 working days of an acute exacerbation.^15^ Some staff felt the COVID-19 pandemic presented an opportunity to better integrate delivery of acute and chronic care by rethinking staff roles within the practice. This might involve supporting staff who previously provided only chronic care to also provide acute care:

*'* *It started out as doing QOF and now it has changed and they are now using me for acute reviews.* *'* (INT7)

However, there was variation between practices, and some participants reported their practice had taken the decision to keep acute and chronic care separate.

#### Contribution: monitoring. Informal appraisal of impact based on experience of implementation

The majority of questionnaire responders reported that they valued the impact that RAC was having on work in their practice (81% agreed or strongly agreed) and felt RAC was worthwhile (81% agreed or strongly agreed). However, interview and focus group participants raised some concerns about the potential impact of RAC on both the patients and the practice based on their experiences of implementation.

Staff reported mixed effects of implementation on patient access. For some patients access had been enhanced, including those for whom RAC was more compatible with their activities of daily living, such as their employment:

*'...* *it has improved the accessibility enormously. I have spoken to asthmatics who felt they never needed to come into the surgery, who have awful DNA* [did not attend] *rate.* *'* (INT7)

For other patients, implementation had negatively impacted their access, including those with poor technology access:

*'* *I think there is a certain cohort who would rather see someone* *… who aren’t technology savvy, older, have hearing impairment* *.'* (INT3)

RAC was felt to be more efficient, with patients less likely to enquire about other issues. However, this was balanced against concerns about the loss of opportunistic assessments. Staff also reported greater fatigue when consulting remotely.

Concerns were raised about the impact of RAC on communication with patients, including the ability to establish rapport. Staff felt there was a greater opportunity for interruptions from the patient’s environment, which could be minimised when face-to-face consulting.

Staff highlighted the increased clinical risk and medicolegal uncertainty associated with RAC:

*'* *I think there is definitely a medico-legal perspective to this as well. It may be* *OK* *to do a virtual clinic when there is no alternative.* *'* (FG5)

#### Contribution: monitoring. Formal appraisal of impact based on measurement

Less than one-third of questionnaire responders felt that their practice had measured or will measure the impact of RAC (31% agreed or strongly agreed). Some staff felt the suspension of QOF targets during the pandemic presented an opportunity to rethink the evaluation of chronic asthma reviews, with a shift from measuring the number of asthma reviews to measurement of patient outcomes including asthma control:

*'* *Initially when QOF came out I saw it as a massive leap forward* *… I would challenge anyone to tell me what outcomes are actually being measured, because I don’t know of any. It’s all about reviews.* *'* (FG3)

While the majority of questionnaire responders felt confident they could modify how they conducted RAC (75% agreed or strongly agreed), there was lack of consensus among interview and focus group participants about how to link appraisal and adaptation. Some practices informally adapted their approach over time based on their experience of implementation including how they selected patients for review. Other practices took a more formal quality improvement approach to implementation, where adaption was guided by measurement.

### Capacity

The incorporation of RAC into its clinical context depends on the capacity of people to cooperate and coordinate their actions. To fully engage with RAC, practices need access to the required technology. Practices were divided by whether their IT system was compatible with software for video consulting and patient messaging software (AccuRx), or they had to rely on the use of telephone for remote consulting:

*'* *I would love to provide that excellent service but I don’t have access to the system to let me do that* *… We need to prioritise getting that level of IT functionality into all practices. It needs to be funded.* *'* (FG9)

Workload impacted the ability of practices to engage with RAC, which was felt to have increased during the pandemic period. Chronic asthma care was particularly affected, with prioritisation of acute care when workload demands increased.

*'* *In my practice because we are under so much pressure because we are down admin staff, we are really prioritising the patients who are unwell.* *'* (FG7)

Patients also need access to the required technology for RAC, including a device for communication (for example, smart phone) and adequate broadband speed:

'*One of the major challenges is the technology at the other end.*' (INT2)

### Capability

The capability of people to enact RAC depends on its workability and integration into everyday practice. Staff not only need access to required technology, but also an understanding of how to use it. Questionnaire responders were divided on whether they had been provided with training on RAC (48% agreed or strongly agreed), and interview and focus group participants highlighted inconsistent access to training on how to use the required technology:

*'* *You are self-taught. I’m sure there is much great functionality which we aren’t using.* *'* (FG3)

Patients also need to be able to use the required technology, and staff felt implementation disadvantaged those who couldn’t. Staff felt ability of patients to engage in RAC was affected by characteristics such as sensory impairment, low literacy, and asthma complexity:

*'* *For the complex and the elderly, I don’t think either of us get what we want from the consultation.* *'* (INT7)

### Potential

To realise the potential of their capability and capacity, staff and patients need to be motivated to engage in RAC. A number of drivers of staff commitment were identified, including the potential impact on patient access and clinical risk, which have already been discussed. Funding appeared to be a key driver for practice commitment:

*'* *You could take it from one practice that they are really engaged right through to* *"* *there’s no QOF so we’re not doing it* *"* *. It is a postcode lottery*.' (INT9)

Staff identified a variety of factors that they felt impacted patient motivation to engage with RAC. Perceived susceptibility appears to be important. For example, staff reported enhanced engagement at the beginning of the COVID-19 pandemic, felt to be owing to the potential increased risk posed by COVID-19 infection for patients with asthma:

*'* *I was quite aware of the worry amongst patients, and we had a real upsurge in the number of requests for inhalers.* *'* (FG5)

Social norms in relation to face-to-face consulting for asthma also appeared to be important. Staff reported that some patients viewed RAC as ‘box-ticking’, and there was resistance from some patients to the adoption of video consulting:

*'* *When I speak to people they say* *,* *"* *Can I not just speak to you on the phone? I can’t be bothered messing about with a video consultation* *"* *.* *'* (FG9)

For other patients, the greater compatibility of RAC with usual activities of daily living increased their ability and motivation to engage, including those with family or employment commitments, which make attendance at the practice during working hours challenging:

*'* *They didn’t have to take time out of their day to come down.* *'* (INT5)

## Discussion

### Summary

The COVID-19 pandemic has simultaneously provided the conditions to make implementation of RAC necessary, yet challenging. While being a key driver for RAC implementation, the pandemic also increased practice workload and reduced the financial incentives to provide chronic asthma care.

This study identified 10 themes to describe and explain the contribution of general practice staff to implementation of RAC. Team members had to identify novel alternatives to in-person review. Having a practice champion, to drive implementation forward and engage other practice staff, was important. Patients, healthcare professionals, and practices varied on many characteristics that are salient to the implementation of RAC, including practice characteristics such as workload, staffing, and access to required technology and training, and patient characteristics such as technology access and/or experience, literacy, and sensory deficits.

The rapid and reactive implementation of RAC in response to pandemic restrictions appears to have disproportionately impacted certain patients and practices, and without appropriate adaptation and monitoring, risks exacerbating existing healthcare inequalities, including those related to access to care.^16^

### Strengths and limitations

This mixed-methods study has a strong theoretical underpinning and highlighted the value of integrating quantitative and qualitative data sources. The extended NPT domains facilitated examination of contextual factors impacting implementation. However, there were some challenges in the use of the framework and presentation of findings, particularly owing to the strong links between framework domains leading to coding and thematic overlaps.

The study had input from a range of general practice staff, with consistent recurring themes identified from participants. However, staff who agreed to participate may have represented a group who are more experienced in the use of RAC, or with more positive attitudes towards it.

The patient perspective on RAC was not included for pragmatic reasons, as the aim of the study was to examine implementation from the perspective of frontline healthcare professionals who deliver RAC. Future work is required to validate the findings from patient perspectives.

### Comparison with existing literature

The majority of staff in this study indicated that RAC has a role for asthma, which is consistent with previous studies that established remote consulting for respiratory conditions is an acceptable approach for staff.^17,18^ Previous studies also raised concerns about how remote consulting impacts access for certain patients,^19^ and is more likely to be used by the younger working population.^16^ As staff in this study reported, face-to-face consulting currently appears to remain preferable for patients with complex presentations.^17,19^

A previous study reported that, when properly integrated into the practice IT systems, video consulting is superior to telephone consulting and offers a time-saving alternative, particularly for the working population.^17^ In this study, staff reported resistance by some patients and practices to the use of video consulting even when they had access to the required technology. For the video consulting to be ‘normalised’ for asthma care, these patient and practice norms will need to be addressed, and there will need to be standardised access to the required software.^19,20^

The findings of this study are consistent with the recently published Planning and Evaluating Remote Consultation Services (PERCS) framework,^21^ including the highlighted importance of an organisation’s digital maturity and digital inclusion efforts.

Previous guidance on remote respiratory consultations makes recommendations that are consistent with the present study's findings, emphasising the importance of preparation before consulting, using questionnaires to assess asthma control, and 'tidying up' after consulting by sending any required written information via email or messaging.^22^

### Implications for practice

The experiences of frontline healthcare professionals in this study indicate that RAC has potential benefits in terms of access and effectiveness when implementation integrates seamlessly with face-to-face care for those who want or need it. ‘Work’ can be done to support implementation and integration of RAC into everyday clinical practice. Specifically, practices can do the following: strive to communicate the value, purpose, and possibilities associated with RAC; identify a practice champion; and engage all members of the multidisciplinary practice and wider primary care team. However, this goes beyond ‘work’ that can be done at an individual staff or practice level. Practices need standardised access to equipment, guidance, and training. Guidance is required on an implementation approach, which is evidence-based, safe, and adapts to patient and practice needs to ensure implementation doesn’t further exacerbate existing healthcare inequalities.
